# Optimizer’s dilemma: optimization strongly influences model selection in transcriptomic prediction

**DOI:** 10.1093/bioadv/vbae004

**Published:** 2024-01-24

**Authors:** Jake Crawford, Maria Chikina, Casey S Greene

**Affiliations:** Genomics and Computational Biology Graduate Group, Perelman School of Medicine, University of Pennsylvania, Philadelphia, PA 19104, United States; Department of Computational and Systems Biology, School of Medicine, University of Pittsburgh, Pittsburgh, PA 15260, United States; Department of Biomedical Informatics, University of Colorado School of Medicine, Aurora, CO 80045, United States; Center for Health AI, University of Colorado School of Medicine, Aurora, CO 80045, United States

## Abstract

**Motivation:**

Most models can be fit to data using various optimization approaches. While model choice is frequently reported in machine-learning-based research, optimizers are not often noted. We applied two different implementations of LASSO logistic regression implemented in Python’s scikit-learn package, using two different optimization approaches (coordinate descent, implemented in the liblinear library, and stochastic gradient descent, or SGD), to predict mutation status and gene essentiality from gene expression across a variety of pan-cancer driver genes. For varying levels of regularization, we compared performance and model sparsity between optimizers.

**Results:**

After model selection and tuning, we found that liblinear and SGD tended to perform comparably. liblinear models required more extensive tuning of regularization strength, performing best for high model sparsities (more nonzero coefficients), but did not require selection of a learning rate parameter. SGD models required tuning of the learning rate to perform well, but generally performed more robustly across different model sparsities as regularization strength decreased. Given these tradeoffs, we believe that the choice of optimizers should be clearly reported as a part of the model selection and validation process, to allow readers and reviewers to better understand the context in which results have been generated.

**Availability and implementation:**

The code used to carry out the analyses in this study is available at https://github.com/greenelab/pancancer-evaluation/tree/master/01_stratified_classification. Performance/regularization strength curves for all genes in the Vogelstein *et al.* (2013) dataset are available at https://doi.org/10.6084/m9.figshare.22728644.

## 1 Introduction

Gene expression profiles are widely used to classify samples or patients into relevant groups or categories, both preclinically (Liu *et al.* 2020, Piccolo *et al.* 2022) and clinically (Albain *et al.* 2009, Parker *et al.* 2009). To extract informative gene features and to perform classification, a diverse array of algorithms exist, and different algorithms perform well across varying datasets and tasks (Piccolo *et al.* 2022). Even within a given model class, multiple optimization methods can often be applied to find well-performing model parameters or to optimize a model’s loss function. One commonly used example is logistic regression. The widely used scikit-learn Python package for machine learning (Pedregosa *et al.* 2011) provides two modules for fitting logistic regression classifiers: LogisticRegression, which uses the liblinear coordinate descent method (Fan *et al.* 2008) to find parameters that optimize the logistic loss function, and SGDClassifier, which uses stochastic gradient descent (Bottou 1998) to optimize the same loss function.

Using scikit-learn, we compared the liblinear (coordinate descent) and SGD optimization techniques for two prediction problems using two cancer transcriptomics datasets. We first considered prediction of driver mutation status in tumor samples, across a wide variety of genes implicated in cancer initiation and development (Vogelstein *et al.* 2013). We additionally predicted gene essentiality (dependency) from gene expression in cancer cell lines, across several genes playing different roles in cancer. We applied LASSO (L1-regularized) logistic regression, and tuned the strength of the regularization to compare model selection between optimizers. We found that across a variety of models (i.e. varying regularization strengths), the training dynamics of the optimizers were considerably different: models fit using liblinear tended to perform best at fairly high regularization strengths (100–1000 nonzero features in the model) and overfit easily with low regularization strengths. On the other hand, after tuning the learning rate, models fit using SGD tended to perform well across both higher and lower regularization strengths, and overfitting was less common.

Our results caution against viewing optimizer choice as a “black box” component of machine learning modeling. The observation that LASSO logistic regression models fit using SGD tended to perform well for low levels of regularization, across diverse driver genes, runs counter to conventional wisdom in machine learning for high-dimensional data which generally states that explicit regularization and/or feature selection is necessary. Comparing optimizers or model implementations directly is rare in applications of machine learning for genomics, and our work shows that this choice can affect generalization and interpretation properties of the model significantly. Based on our results, we recommend considering the appropriate optimization approach carefully based on the goals of each individual analysis.

## 2 Methods

### 2.1 TCGA data download and preprocessing

To generate binary mutated/non-mutated gene labels for our machine learning model, we used mutation calls for TCGA Pan-Cancer Atlas samples from MC3 (Ellrott *et al.* 2018) and copy number threshold calls from GISTIC2.0 (Mermel *et al.* 2011). MC3 mutation calls were downloaded from the Genomic Data Commons (GDC) of the National Cancer Institute, at https://gdc.cancer.gov/about-data/publications/pancanatlas. Thresholded copy number calls are from an older version of the GDC data and are available here: https://figshare.com/articles/dataset/TCGA_PanCanAtlas_Copy_Number_Data/6144122. We removed hypermutated samples, defined as two or more standard deviations above the mean non-silent somatic mutation count, from our dataset to reduce the number of false positives (i.e. non-driver mutations). Any sample with either a non-silent somatic variant or a copy number variation (copy number gain in the target gene for oncogenes and copy number loss in the target gene for tumor suppressor genes) was included in the positive set; all remaining samples were considered negative for mutation in the target gene.

RNA sequencing data for TCGA were downloaded from GDC at the same link provided above for the Pan-Cancer Atlas. We discarded non-protein-coding genes and genes that failed to map and removed tumors that were measured from multiple sites. After filtering to remove hypermutated samples and taking the intersection of samples with both mutation and gene expression data, 9074 total TCGA samples remained.

### 2.2 Cancer gene set construction

In order to study mutation status classification for a diverse set of cancer driver genes, we started with the set of 125 frequently altered genes from Vogelstein *et al.* (2013) (all genes from Supplementary Table S2A). For each target gene, in order to ensure that the training dataset was reasonably balanced (i.e. that there would be enough mutated samples to train an effective classifier), we included only cancer types with both: (i) at least 15 of the total samples for the given cancer type are mutated, and (ii) at least 5% of the total samples for the given cancer type are mutated. We refer to these cancer types here as “valid” cancer types. For genes that are not frequently mutated, this occasionally resulted in no valid cancer types, and we dropped these genes from the analysis. Out of the 125 genes originally listed in the Vogelstein *et al.* cancer gene set, we retained 84 target genes.

### 2.3 Mutation status prediction classifier setup and data splitting

We trained logistic regression classifiers to predict whether or not a given sample had a mutational event in a given target gene, using gene expression features as explanatory variables or signatures of mutation. Our models were trained on gene expression data as features, or predictor variables (*X*, 16 148 input genes from pre-processed TCGA RNA-seq data). The response/target variable used (*y*) was the presence or absence of a mutation in a target gene, generated for each sample as described in the “Data download and preprocessing” section. Based on our previous work, gene expression is generally effective for this problem across many target genes, so we limited our analyses in this study to this data type (Crawford *et al.* 2022). To control for varying mutation burden per sample and to adjust for potential cancer type-specific expression patterns, we included one-hot encoded cancer type and log_10_(sample mutation count) in the model as covariates, in addition to the gene expression features.

To compare model selection across optimizers on a consistent set of held-out data, we first split the “valid” cancer types into train (75%) and test (25%) sets. We then split the training data into “subtrain” (66% of the training set) data to train the model on, and “holdout” (33% of the training set) data to perform model selection, i.e. to use to select the best-performing regularization parameter, and the best-performing learning rate for SGD in the cases where multiple learning rates were considered. In each case, these splits were stratified by cancer type, i.e. each split had as close as possible to equal proportions of each cancer type included in the dataset for the given driver gene.

### 2.4 LASSO parameter range selection and comparison between optimizers

Since gene expression datasets tend to have many dimensions and comparatively few samples, we used a LASSO penalty to perform feature selection (Tibshirani 1996). LASSO logistic regression has the advantage of generating sparse models (some or most coefficients are 0), as well as having a single tunable hyperparameter which can be easily interpreted as an indicator of regularization strength, or model complexity. The scikit-learn implementations of coordinate descent (in liblinear/LogisticRegression) and stochastic gradient descent (in SGDClassifier) use slightly different parameterizations of the LASSO regularization strength parameter. liblinear’s logistic regression solver optimizes the following loss function:

$$\hat{w}={\text{argmin}}_{w}\;\left(C\cdot \ell \left(X,y;w\right)\right)+||w{\left|\right|}_{1}$$

where $\ell \left(X,y;w\right)$ denotes the negative log-likelihood of the observed data $\left(X,y\right)$ given a particular choice of feature weights w. SGDClassifier optimizes the following loss function:

$$\hat{w}={\text{argmin}}_{w}\;\ell \left(X,y;w\right)+\alpha ||w{\left|\right|}_{1}$$

which is equivalent with the exception of the LASSO parameter which is formulated slightly differently, as α=1/C. The result of this slight difference in parameterization is that liblinearC values vary inversely with regularization strength (higher values = less regularization, or greater model complexity) and SGDClassifierα values vary directly with regularization strength (lower values = less regularization, or greater model complexity).

For the liblinear optimizer, we trained models using C values evenly spaced on a logarithmic scale between (10^−3^,10^7^); i.e. the output of numpy.logspace(-3, 7, 21). For the SGD optimizer, we trained models using the inverse range of α values between (10^−7^, 10^3^), or numpy.logspace(-7, 3, 21). These hyperparameter ranges were intended to give evenly distributed coverage across genes that included “underfit” models (predicting only the mean or using very few features, poor performance on all datasets), “overfit” models (performing perfectly on training data but comparatively poorly on cross-validation and test data), and a wide variety of models in between that typically included the best fits to the cross-validation and test data.

For ease of visual comparison in our figures, we plot the SGD α parameter directly, and the liblinearC parameter inversely (i.e. 1/C). This orients the x-axes of the relevant plots in the same direction: lower values represent lower regularization strength or higher model complexity, and higher values represent higher regularization strength or lower model complexity, for both optimizers.

### 2.5 SGD learning rate selection

scikit-learn’s SGDClassifier provides four built-in approaches to learning rate scheduling: constant (a single, constant learning rate), optimal (a learning rate with an initial value selected using a heuristic based on the regularization parameter and the data loss, that decreases across epochs), invscaling (a learning rate that decreases exponentially by epoch), and adaptive (a learning rate that starts at a constant value, which is divided by five each time the training loss fails to decrease for five straight epochs). The optimal learning rate schedule is used by default.

When we compared these four approaches, we used a constant learning rate of 0.0005, and an initial learning rate of 0.1 for the adaptive and invscaling schedules. We also tested a fifth approach that we called “constant_search,” in which we tested a range of constant learning rates in a grid search on a validation dataset, then evaluated the model on the test data using the best-performing constant learning rate by validation AUPR. For the grid search, we used the following range of constant learning rates: {0.00001, 0.0001, 0.001, 0.01}. Unless otherwise specified, results for SGD in the main paper figures used the constant_search approach, which performed the best in our comparison between schedulers.

### 2.6 DepMap gene essentiality prediction

To generate binary essential/not essential gene labels for cell lines, we used the data available on the Cancer Dependency Map (DepMap) download portal at https://depmap.org/portal/download/all/. Essentiality information for each gene perturbation was downloaded in the CRISPRGeneDependency.csv file (version 23Q2), and gene expression information was downloaded in the CCLE_expression.csv file (version 22Q2). We thresholded the gene dependency probabilities into the top 20% (most likely to be a dependency on the given gene in the given cell type) and bottom 80%, assigning a 1 label to the former and a 0 label to the latter. We integrated the gene dependency data with the gene expression data using the DepMap_ID identifier, and dropped any cell lines that were not present in both datasets. We preprocessed the gene expression data using the same steps as for the TCGA data, resulting in a total of 17931 gene features. We applied the same cancer type filters and cross-validation setup stratified by cancer type as for the TCGA data as well.

For benchmarking of essentiality prediction and comparison of optimization strategies, we aimed to choose several genes with diverse functions, but our goal was not to explore the space of gene perturbations completely since other studies have addressed this, e.g. Dempster *et al.* (2020). We chose 5 oncogenes (*BRAF*, *EGFR*, *ERBB2*, *KRAS*, *PIK3CA*) which have been documented as examples of “oncogene addiction”; i.e. cancer cells with a mutation in these genes are dependent on the mutation, and when it is reversed or removed this is lethal. We additionally chose five genes with known synthetic lethal relationships in a subset of cancers, some of which have targeted therapies in clinical trials or in current clinical use: *PARP1* (Feng *et al.* 2015), *RAD52* (Huang *et al.* 2016), *POLQ* (Feng *et al.* 2019), *USP1* (Simoneau *et al.* 2022), and *PRMT5* (Wei *et al.* 2020). Finally, we chose three more genes that were highlighted in a DepMap study (Tsherniak *et al.* 2017) as having “differential dependencies” across cell lines: *PTPN11*, *MDM4*, and *CYFIP1*.

## 3 Results

### 3.1 liblinear and SGD LASSO models perform comparably, but liblinear is sensitive to regularization strength

For each of 84 driver genes from the Vogelstein *et al.* (2013) paper, we trained models to predict mutation status (presence or absence) from RNA-seq data, derived from the TCGA Pan-Cancer Atlas. Gene expression signatures that distinguish mutated from wild-type samples have been previously validated in multiple cancer driver genes and pathways of interest (Knijnenburg *et al.* 2018, Way *et al.* 2018, Kang *et al.* 2020) and benchmarked broadly across genes and data types (Way *et al.* 2020, Crawford *et al.* 2022), and the resulting signatures or classifiers can be used to identify patients with atypical alterations or susceptibilities to targeted therapy (Haan *et al.* 2019, Bakhtiar *et al.* 2022, Li *et al.* 2022). For each optimizer, we trained LASSO logistic regression models across a variety of regularization parameters (see Section 2 for parameter range details), achieving a variety of different levels of model sparsity (Supplementary Fig. S1). We repeated model fitting/evaluation across four cross-validation splits × two replicates (random seeds) for a total of eight different models per parameter. Cross-validation splits were stratified by cancer type.

**Figure 1.**
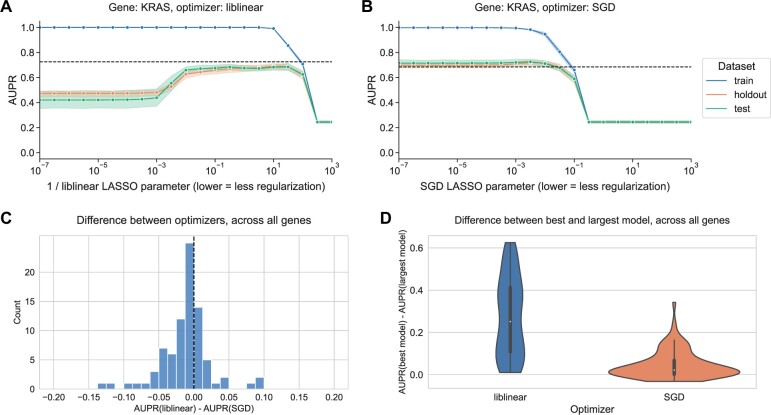
(A) Performance versus inverse regularization parameter for KRAS mutation status prediction, using the liblinear coordinate descent optimizer. Dotted lines indicate top performance value of the opposite optimizer. (B) Performance versus regularization parameter for KRAS mutation status prediction, using the SGD optimizer. “Holdout” dataset is used for SGD learning rate selection, “test” data are completely held out from model selection and used for evaluation. (C) Distribution of performance difference between best-performing model for liblinear and SGD optimizers, across all 84 genes in Vogelstein driver gene set. Positive numbers on the *x*-axis indicate better performance using liblinear, and negative numbers indicate better performance using SGD. (D) Distribution of performance difference between best-performing model and largest (least regularized) model, for liblinear and SGD, across all 84 genes. Smaller numbers on the *y*-axis indicate less overfitting, and larger numbers indicate more overfitting.

Previous work has shown that pan-cancer classifiers of Ras mutation status are accurate and biologically informative (Way *et al.* 2018). We first evaluated models for KRAS mutation prediction. As model complexity increases (more nonzero coefficients) for the liblinear optimizer, we observed that performance increases then decreases, corresponding to overfitting for high model complexities/numbers of nonzero coefficients (Fig. 1A). On the other hand, for the SGD optimizer, we observed consistent performance as model complexity increases, with models having no nonzero coefficients performing comparably to the best (Fig. 1B).

In this case, top performance for SGD (a regularization parameter of 3.16 × 10^−3^) is slightly better than top performance for liblinear (a regularization parameter of 1/3.16 × 10^2^): we observed a mean test AUPR of 0.725 for SGD versus mean AUPR of 0.685 for liblinear.

To determine how relative performance trends with liblinear tend to compare across the genes in the Vogelstein dataset at large, we looked at the difference in performance between optimizers for the best-performing models for each gene (Fig. 1C). The distribution is centered around 0 and more or less symmetrical, suggesting that across the gene set, liblinear and SGD tend to perform comparably to one another. We saw that for 58/84 genes, performance for the best-performing model was better using SGD than liblinear, and for the other 25 genes performance was better using liblinear. In order to quantify whether the overfitting tendencies (or lack thereof) also hold across the gene set, we plotted the difference in performance between the best-performing model and the largest (least regularized) model; classifiers with a large difference in performance exhibit strong overfitting, and classifiers with a small difference in performance do not overfit (Fig. 1D). For SGD, the least regularized models tend to perform comparably to the best-performing models, whereas for liblinear the distribution is wider suggesting that overfitting is more common.

### 3.2 SGD is sensitive to learning rate selection

**Figure 2.**
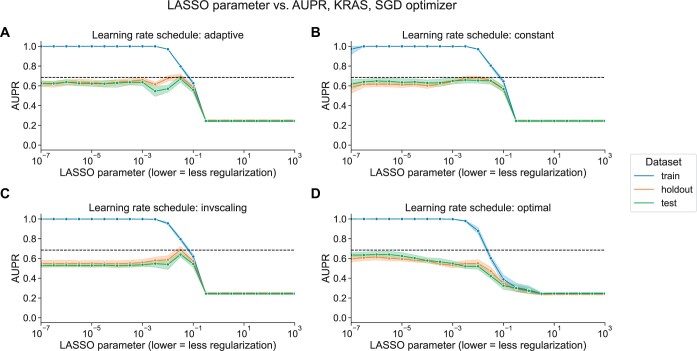
(A) Performance versus regularization parameter for KRAS mutation prediction, using SGD optimizer with adaptive learning rate scheduler. Dotted line indicates top performance value using liblinear, from Fig. 1A. (B) Performance versus regularization parameter, using SGD optimizer with constant learning rate scheduler and a learning rate of 0.0005. (C) Performance versus regularization parameter, using SGD optimizer with inverse scaling learning rate scheduler. (D) Performance versus regularization parameter, using SGD optimizer with “optimal” learning rate scheduler.

The SGD results shown in Fig. 1 select the best-performing learning rate using a grid search on the holdout dataset, independently for each regularization parameter. We also compared against other learning rate scheduling approaches implemented in scikit-learn (see Section 2 for implementation details and grid search specifications). For KRAS mutation prediction, we observed that the choice of initial learning rate and scheduling approach affects performance significantly, and other approaches to selecting the learning rate performed poorly relative to liblinear (black dotted lines in Fig. 2) and to the grid search.

We did not observe an improvement in performance over liblinear or the grid search for learning rate schedulers that decrease across epochs (Fig. 2A, C, and D), nor did we see comparable performance when we selected a single constant learning rate for all levels of regularization without the preceding grid search (Fig. 2B). Notably, scikit-learn’s default “optimal” learning rate schedule performed relatively poorly for this problem, suggesting that tuning the learning rate and selecting a well-performing scheduler is a critical component of applying SGD successfully for this problem (Fig. 2D). We observed similar trends across all genes in the Vogelstein gene set, with other learning rate scheduling approaches performing poorly in aggregate relative to both liblinear and SGD with the learning rate grid search (Supplementary Fig. S2).

### 3.3 liblinear and SGD result in different models, with varying loss dynamics

**Figure 3.**
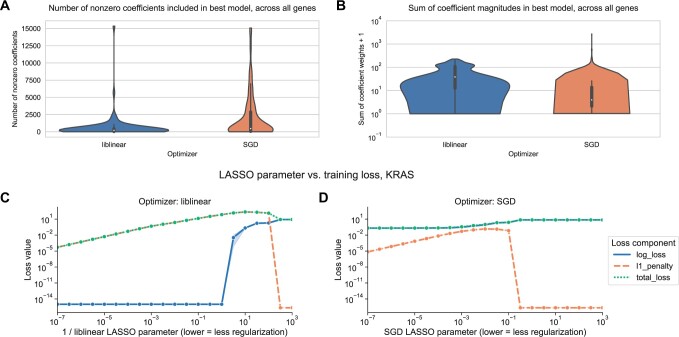
(A) Distribution across genes of the number of nonzero coefficients included in best-performing LASSO logistic regression models. Violin plot density estimations are clipped at the ends of the observed data range, and boxes show the median/IQR. (B) Distribution across genes of the sum of model coefficient weights for best-performing LASSO logistic regression models. (C) Decomposition of loss function for models fit using liblinear across regularization levels. Zero values on the *y*-axis are rounded up to machine epsilon; i.e. 2.22 × 10^–16^. (D) Decomposition of loss function for models fit using SGD across regularization levels. Zero values on the *y*-axis are rounded up to machine epsilon; i.e. 2.22 × 10^–16^.

We sought to determine whether there was a difference in the sparsity of the models resulting from the different optimization schemes. In general across all genes, the best-performing SGD models mostly tend to have many nonzero coefficients, but with a distinct positive tail, sometimes having few nonzero coefficients. In contrast, the liblinear models are generally sparser with fewer than 2500 nonzero coefficients, out of ∼16 100 total input features, and a much narrower tail (Fig. 3A). The sum of the coefficient magnitudes, however, tends to be smaller on average across all levels of regularization for SGD than for liblinear (Fig. 3B). This effect is less pronounced for the other learning rate schedules shown in Fig. 2, with the other options resulting in larger coefficient magnitudes (Supplementary Fig. S3).

These results suggest that the models fit by liblinear and SGD navigate the tradeoff between bias and variance in slightly different ways: liblinear tends to produce sparser models (more zero coefficients) as regularization increases, but if the learning rate is properly tuned, SGD coefficients tend to have smaller overall magnitudes as regularization increases.

We also compared the components of the loss function across different levels of regularization between optimizers. The LASSO logistic regression loss function can be broken down into a data-dependent component (the log-loss) and a parameter magnitude dependent component (the L1 penalty), which are added to get the total loss that is minimized by each optimizer; see Section 2 for additional details. As regularization strength decreases for liblinear, the data loss collapses to near 0, and the L1 penalty dominates the overall loss (Fig. 3C). For SGD, on the other hand, the data loss decreases slightly as regularization strength decreases but remains relatively high (Fig. 3D). Other SGD learning rate schedules have similar loss curves to the liblinear results, although this does not result in improved classification performance (Supplementary Fig. S4).

### 3.4 Gene essentiality prediction in cancer cell lines yields similar results

**Figure 4.**
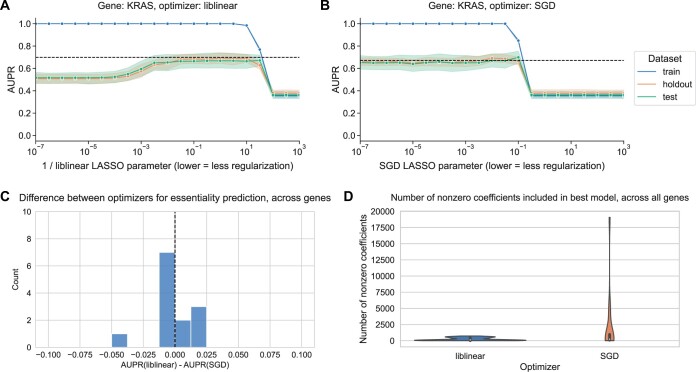
(A) Performance versus inverse regularization parameter for KRAS gene essentiality prediction, using the liblinear coordinate descent optimizer. (B) Performance versus regularization parameter for KRAS gene essentiality prediction, using the SGD optimizer. “Holdout” dataset is used for SGD learning rate selection, “test” data are completely held out from model selection and used for evaluation. (C) Distribution of performance difference between best-performing model for liblinear and SGD optimizers, across all 13 genes in gene essentiality prediction set. Positive numbers on the *x*-axis indicate better performance using liblinear, and negative numbers indicate better performance using SGD. (D) Distribution across 13 genes of the number of nonzero coefficients included in best-performing LASSO logistic regression models for essentiality prediction.

As a complementary problem to mutation status prediction in human tumor samples, we binarized gene essentiality probabilities from the Cancer Dependency Map (DepMap) into the top 20% and bottom 80%, then used the same stratified cross-validation setup as before to predict whether or not held-out cell lines belonged to the top 20% using cell line gene expression data. We evaluated this for 13 genes, with a variety of cancer functions: 5 oncogenes (*BRAF*, *EGFR*, *ERBB2*, *KRAS*, *PIK3CA*) where “oncogene addiction” has been observed, 5 genes (*PARP1*, *RAD52*, *POLQ*, *USP1*, *PRMT5*) with known synthetic lethal relationships, and 3 genes (*PTPN11*, *MDM4*, *CYFIP1*) labeled as having “differential dependencies” in a study of gene dependencies in DepMap (Tsherniak *et al.* 2017); additional detail in Section 2. For modeling *KRAS* perturbation, we saw a similar trend in the cell line data as in the mutation prediction example, where liblinear overfits for high model complexities (Fig. 4A) and SGD is more resistant to overfitting (Fig. 4B).

Although performance across the other selected gene perturbations varied considerably (Supplementary Fig. S5), we saw largely similar trends across other genes where models performed well, with the exception of *ERBB2* which did tend to overfit for SGD as well as liblinear (Supplementary Fig. S6).

Across all 13 genes, when we compared the best-performing models for liblinear and SGD, we did not see a systematic advantage for either optimizer, matching the results of the comparison across genes for mutation status prediction (Fig. 4C). Similar to the pattern in Fig. 3A, for gene essentiality prediction we also observed that liblinear-optimized models tended to be smaller on average than models optimized by SGD, with a relatively condensed distribution for liblinear on the order of hundreds to thousands of genes, but a “long tail” for SGD extending to models with tens of thousands of genes (Fig. 4D). In general, these data suggest that the tradeoff between optimizers yields comparable results, and comparable recommendations, for a related classification problem on a distinct cancer transcriptomics dataset.

## 4 Discussion

Our work shows that optimizer choice presents tradeoffs in model selection for cancer transcriptomics. We observed that LASSO logistic regression models for mutation status prediction and gene essentiality prediction fit using stochastic gradient descent were highly sensitive to learning rate tuning, but they tended to perform robustly across diverse levels of regularization and sparsity. Coordinate descent implemented in liblinear did not require learning rate tuning, but generally performed best for a narrow range of fairly sparse models, overfitting as regularization strength decreased. Tuning of regularization strength for liblinear, and learning rate (and regularization strength to a lesser degree) for SGD, are critical steps which must be considered as part of analysis pipelines. The sensitivity we observed to these details highlights the importance of reporting exactly what optimizer was used, and how the relevant hyperparameters were selected, in studies that use machine learning models for transcriptomic data. We recommend that both researchers and reviewers emphasize consideration of these steps, and transparency in reporting them.

To our knowledge, the phenomenon we observed with SGD has not been documented in other applications of machine learning to genomic or transcriptomic data. In recent years, however, the broader machine learning research community has identified and characterized implicit regularization for SGD in many settings, including overparameterized or feature-rich problems as is often the case in transcriptomics (Dauber *et al.* 2020, Zou *et al.* 2021a,b). The resistance we observed of SGD-optimized models to decreased performance on held-out data as model complexity increases is often termed “benign overfitting”: overfit models, in the sense that they fit the training data perfectly and perform worse on the test data, can still outperform models that do not fit the training data as well or that have stronger explicit regularization. Benign overfitting has been attributed to optimization using SGD (Zhang *et al.* 2021, Zou et al. 2021a), and similar patterns have been observed for both linear models and deep neural networks (Zhang *et al.* 2017, Bartlett *et al.* 2020).

Existing gene expression prediction benchmarks and pipelines typically use a single model implementation, and thus a single optimizer. We recommend thinking critically about optimizer choice, but this can be challenging for researchers that are inexperienced with machine learning or unfamiliar with how certain models are optimized under the hood. For example, R’s glmnet package uses a cyclical coordinate descent algorithm to fit logistic regression models (Friedman *et al.* 2010), which would presumably behave similarly to liblinear, but this is somewhat opaque in the glmnet documentation itself. Increased transparency and documentation in popular machine learning packages with respect to optimization, especially for models that are difficult to fit or sensitive to hyperparameter settings, would benefit new and unfamiliar users.

Related to what we see in our SGD-optimized models, there exist other problems in gene expression analysis where using all available features is comparable to, or better than, using a subset. For example, using the full gene set improves correlations between preclinical cancer models and their tissue of origin, as compared to selecting genes based on variability or tissue-specificity (Williams *et al.* 2023). On the other hand, in a broader study than ours of cell line viability prediction from gene expression profiles across 100 gene perturbations and 5 different datasets, selecting features by Pearson correlation improves performance over using all features, similar to our liblinear classifiers (Dempster *et al.* 2020). In future work, it could be useful to explore if the coefficients found by liblinear and SGD emphasize the same pathways or functional gene sets, or if there are patterns to which mutation status classifiers (or other cancer transcriptomics classifiers) perform better with more/fewer nonzero coefficients.

Similarly, it would be interesting to explore in more detail the degree to which sample size, particularly the proportion of samples containing a particular driver mutation, affects model performance and optimizer dynamics. Although we observed in previous work that mutation status classifiers for cancer-related genes tend to outperform classifiers for random genes with similar mutation proportions (Crawford *et al.* 2022), our dataset of cancer genes is likely enriched for genes that are commonly mutated across cancer types, rather than specifically having a driver role in one or a few cancers. A more in-depth study of cancer type-specific drivers could identify more localized patterns in which optimizer performs best and how this may correlate with the dimensions of the dataset, which could be averaged over or smoothed out by our pan-cancer approach in this study.

## Supplementary Material

vbae004_Supplementary_Data

## Data Availability

The data analyzed during this study were previously published as part of the TCGA Pan-Cancer Atlas project (Weinstein *et al.* 2013), and are available from the NIH NCI Genomic Data Commons (GDC). The scripts used to download and preprocess the datasets for this study are available at https://github.com/greenelab/pancancer-evaluation/tree/master/00_process_data, and the code used to carry out the analyses in this study is available at https://github.com/greenelab/pancancer-evaluation/tree/master/01_stratified_classification, both under the open-source BSD 3-clause license. Tables showing mutation counts and proportion of samples mutated for each gene and cancer type in the dataset are available on Figshare at https://doi.org/10.6084/m9.figshare.24442624, under a CC0 license. Equivalent versions of Fig. 1A and B for all 84 genes in the Vogelstein *et al.* (2013) gene set are available on Figshare at https://doi.org/10.6084/m9.figshare.22728644, under a CC0 license. This manuscript was written using Manubot (Himmelstein *et al.* 2019) and is available on GitHub at https://github.com/greenelab/optimizer-manuscript under the CC0-1.0 license. This research was supported in part by the University of Pittsburgh Center for Research Computing through the resources provided. Specifically, this work used the HTC cluster, which is supported by NIH award number S10OD028483.
