# Supplementary material for: Optimizer’s dilemma: optimization strongly influences model selection in transcriptomic prediction
Source: Bioinform Adv. 2024 Jan 24;4(1):vbae004. doi: 10.1093/bioadv/vbae004 (PMC10822580; doi:10.1093/bioadv/vbae004)
Supplement: vbae004_Supplementary_Data [file vbae004_supplementary_data.pdf]

Supplementary Material

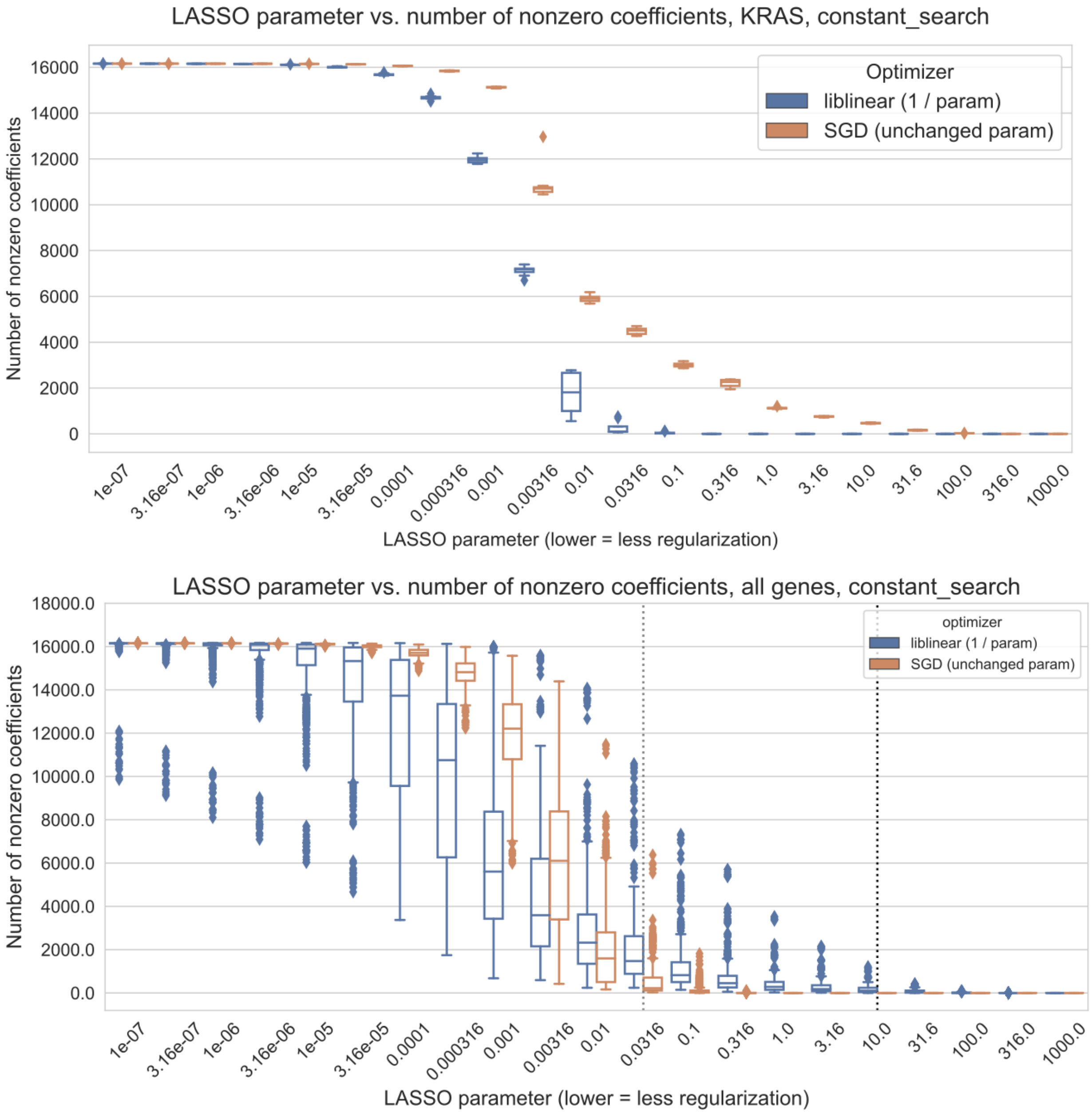

Figure S1: Number of nonzero coefficients (model sparsity) across varying regularization parameter settings for KRAS mutation prediction using SGD and `liblinear` optimizers, and averaged across all genes for both optimizers. In the “all genes” plot, the black dotted line shows the median parameter selected for `liblinear`, and the grey dotted line shows the median parameter selected for SGD.

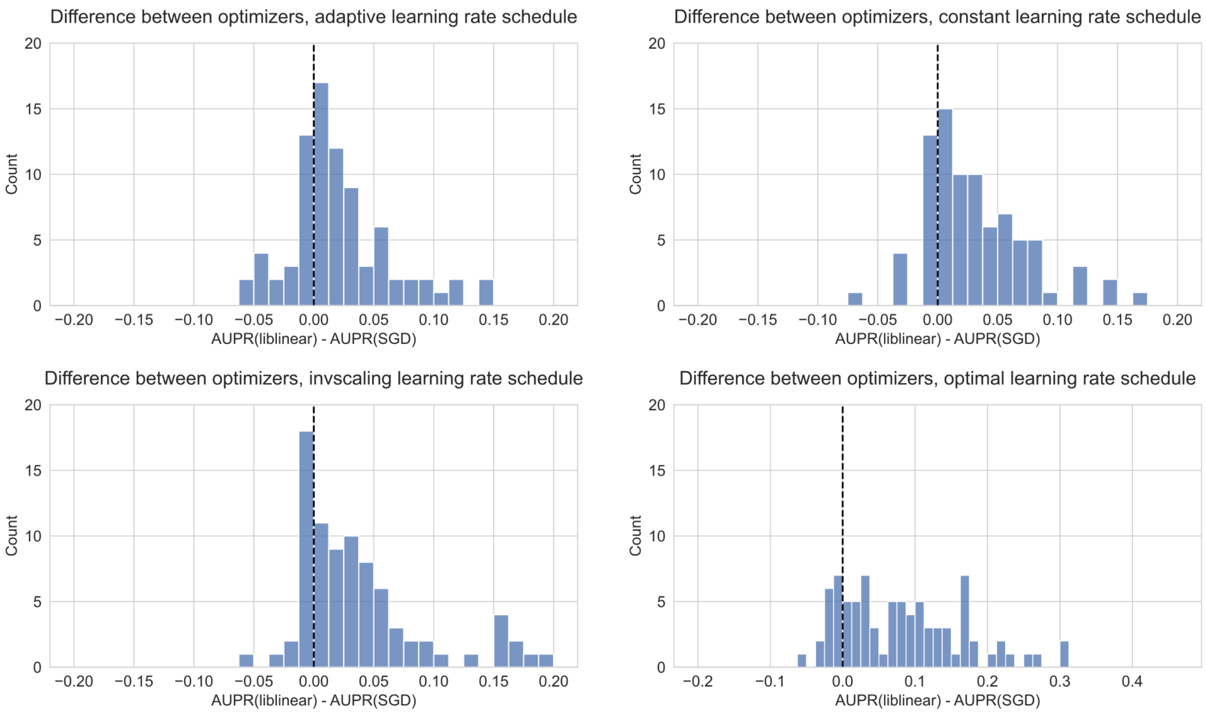

Figure S2: Distribution of performance difference between best-performing model for `liblinear` and SGD optimizers, across all 84 genes in Vogelstein driver gene set, for varying SGD learning rate schedulers. Positive numbers on the x-axis indicate better performance using `liblinear`, and negative numbers indicate better performance using SGD.

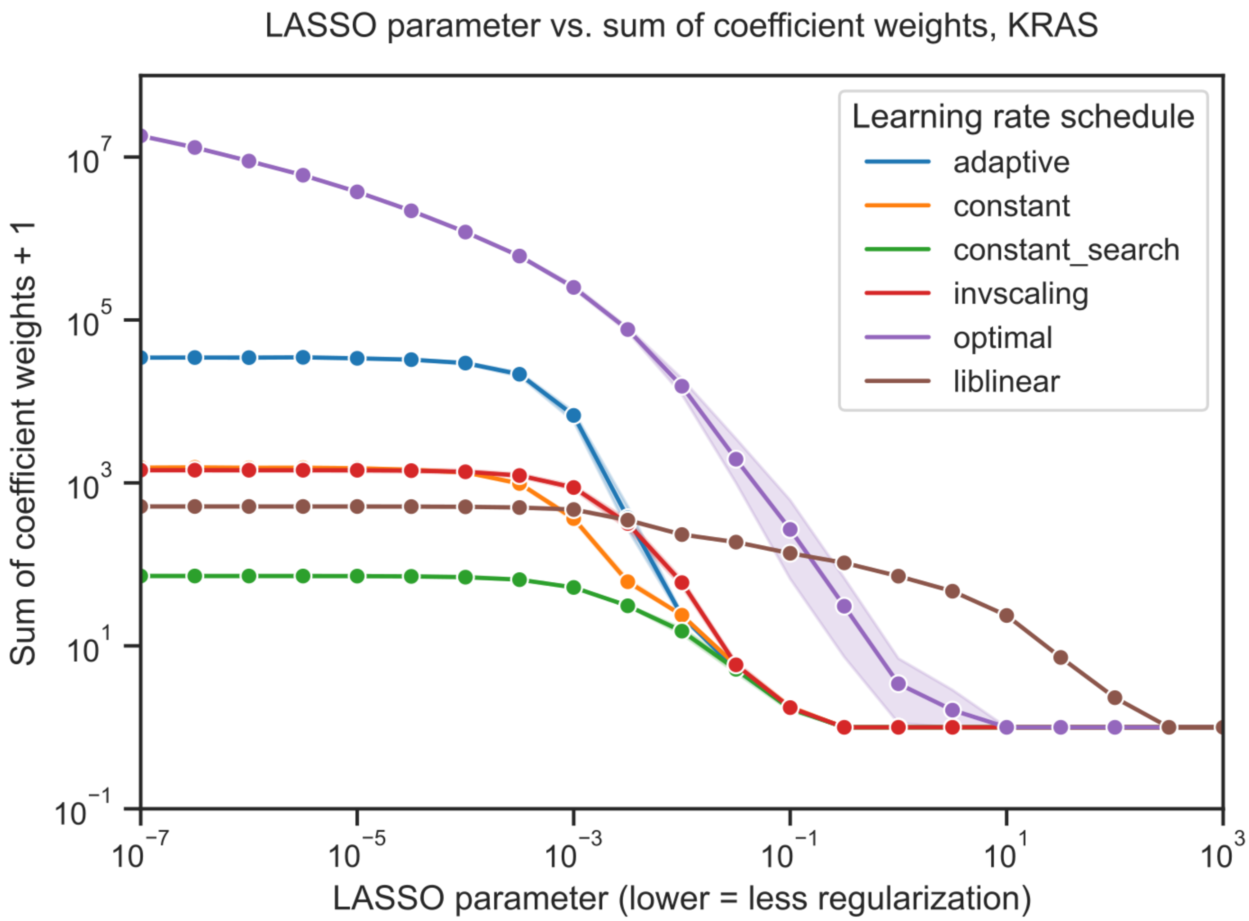

Figure S3: Sum of absolute value of coefficients + 1 for KRAS mutation prediction using SGD and `liblinear` optimizers, with varying learning rate schedules for SGD. Similar to the figures in the main paper, the `liblinear` x-axis represents the inverse of the C regularization parameter; SGD x-axes represent the untransformed α parameter.

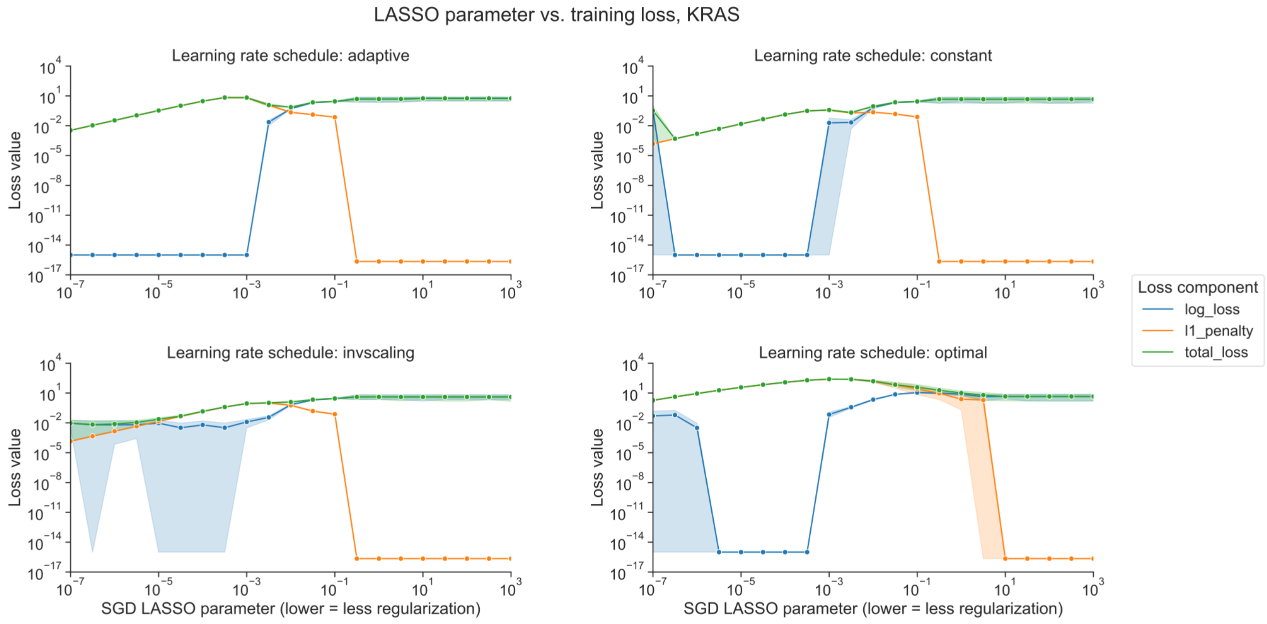

Figure S4: Decomposition of loss function into data loss and L1 penalty components for KRAS mutation prediction using SGD optimizer, across regularization levels, using varying learning rate schedulers. 0 values on the y-axis are rounded up to machine epsilon, i.e. 2.22 x 10^{-16}.

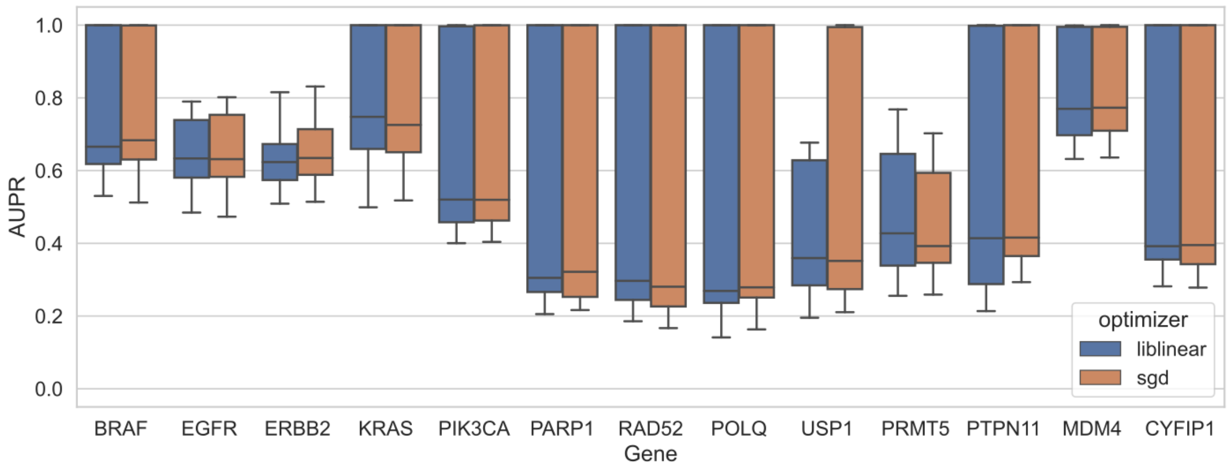

Figure S5: Performance on held-out data for DepMap gene essentiality prediction from cell line gene expression for 13 different genes, across cross-validation splits.

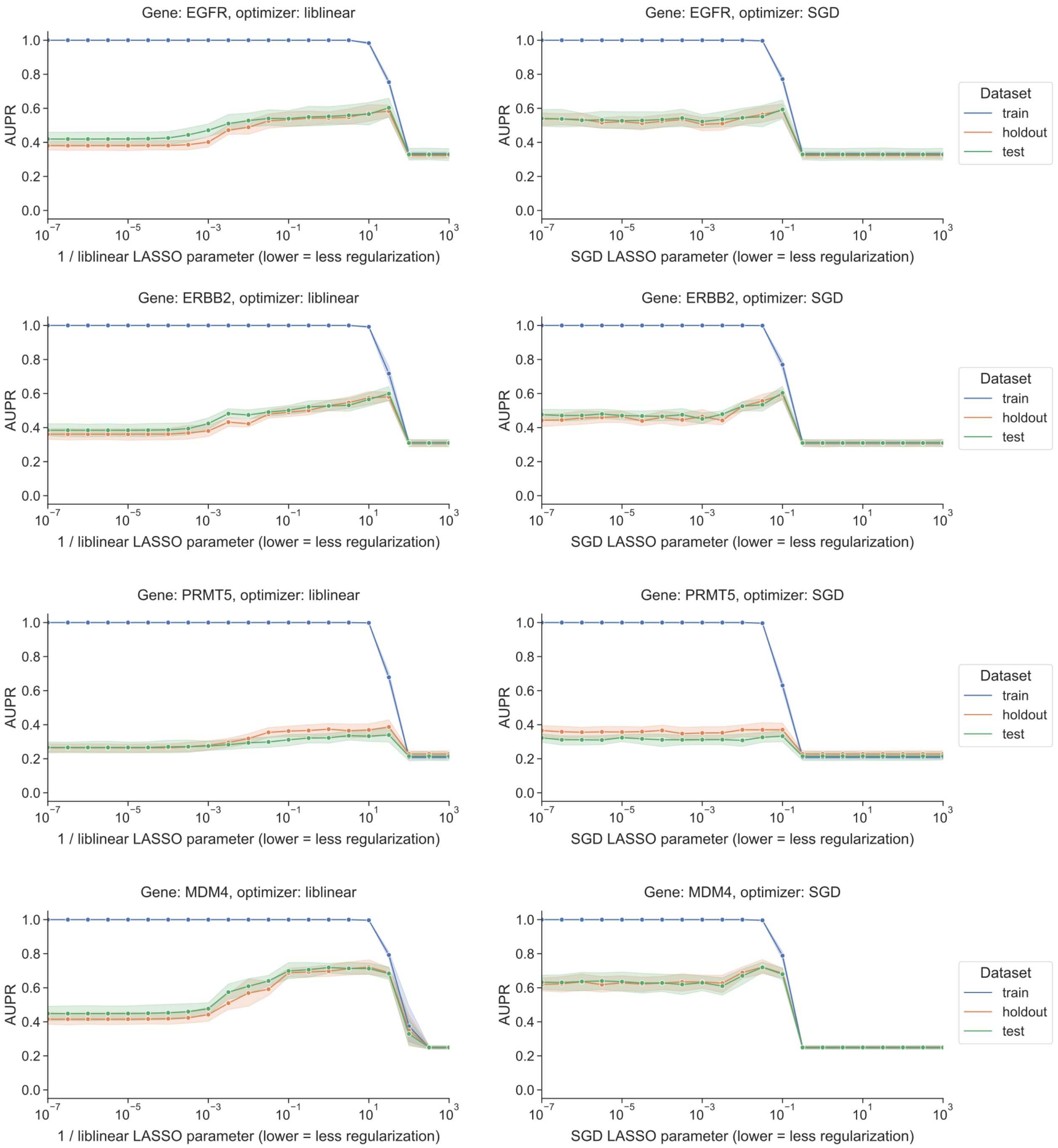

Figure S6: Performance vs. regularization parameter for DepMap gene essentiality prediction, for `liblinear` coordinate descent and SGD using a search over constant learning rates. "Holdout" dataset is used for SGD learning rate selection, "test" data is completely held out from model selection and used only for evaluation.
